# Vacuolar processing enzyme in plant programmed cell death

**DOI:** 10.3389/fpls.2015.00234

**Published:** 2015-04-09

**Authors:** Noriyuki Hatsugai, Kenji Yamada, Shino Goto-Yamada, Ikuko Hara-Nishimura

**Affiliations:** ^1^Department of Plant Biology, Microbial and Plant Genomics Institute, University of MinnesotaSt. Paul, MN, USA; ^2^Department of Botany, Graduate School of Science, Kyoto UniversityKyoto, Japan

**Keywords:** asparaginyl endopeptidase (AEP), caspase 1, hypersensitive cell death, legumain, programmed cell death, senescence, vacuolar collapse, vacuolar processing enzyme (VPE)

## Abstract

Vacuolar processing enzyme (VPE) is a cysteine proteinase originally identified as the proteinase responsible for the maturation and activation of vacuolar proteins in plants, and it is known to be an ortholog of animal asparaginyl endopeptidase (AEP/VPE/legumain). VPE has been shown to exhibit enzymatic properties similar to that of caspase 1, which is a cysteine protease that mediates the programmed cell death (PCD) pathway in animals. Although there is limited sequence identity between VPE and caspase 1, their predicted three-dimensional structures revealed that the essential amino-acid residues for these enzymes form similar pockets for the substrate peptide YVAD. In contrast to the cytosolic localization of caspases, VPE is localized in vacuoles. VPE provokes vacuolar rupture, initiating the proteolytic cascade leading to PCD in the plant immune response. It has become apparent that the VPE-dependent PCD pathway is involved not only in the immune response, but also in the responses to a variety of stress inducers and in the development of various tissues. This review summarizes the current knowledge on the contribution of VPE to plant PCD and its role in vacuole-mediated cell death, and it also compares VPE with the animal cell death executor caspase 1.

## Introduction

Unlike necrotic cell death, which results from accidental and physical damage, programmed cell death (PCD) is a genetically regulated physiological process of cell suicide that is integral to the development and survival of eukaryotes. In animal cells, apoptosis, the most characterized form of PCD, is executed by a family of highly conserved proteinases known as caspases (Cohen, 1997). Caspases are cysteine proteases that cleave their substrates after P_1_ position aspartic acid residues. The amino acids preference at the P_2_–P_4_ positions differs among caspase family members, including the peptide sequence of YVAD for caspase 1, DEVD for caspase 3, VEID and VKMD for caspase 6, and IETD for caspase 8.

In 1998, a proteolytic activity toward a synthetic caspase-1 substrate (N-acetyl-YVAD-MCA) was detected in tobacco plants and the activity was required for bacterially induced PCD (del Pozo and Lam, 1998). This was the first research to suggest that the presence of a caspase-like proteinase was related to plant cell death. However, because no caspase orthologs had been identified in plant genomes, the plant proteinase catalyzing the caspase-1 substrate was unknown until the vacuolar processing enzyme (VPE) was identified as a plant proteinase with a caspase-1-like activity (Hatsugai et al., 2004).

Homology searches have uncovered the existence of several metacaspases in plants that contain the caspase-conserved domains, and they have been proposed to play a role in plant PCD (Uren et al., 2000; Coll et al., 2011) (Table 1). However, metacaspases lack the aspartic acid specificity of caspases, and they cleave their substrates after arginine and lysine residues (Tsiatsiani et al., 2011). In the past decade, several groups have identified plant proteinases that exhibit caspase-like activities responsible for PCD (Table 1). The subtilisin-like serine proteases, saspase, and phytaspase, have caspase-6-like activities that are associated with pathogen-induced PCD in *Avena sativa* (Coffeen and Wolpert, 2004; Chichkova et al., 2010; Vartapetian et al., 2011). Additionally, in *Arabidopsis thaliana*, the 26S proteasome β subunit PBA1 catalyzes caspase-3 substrates and mediates PCD during bacterial infection (Hatsugai et al., 2009; Hatsugai and Hara-Nishimura, 2010; Hara-Nishimura and Hatsugai, 2011) and xylem development (Han et al., 2012). Plant proteinases, as related to caspase-like activities, have been extensively reviewed (Lam and del Pozo, 2000; Woltering et al., 2002; Sanmartin et al., 2005; Bonneau et al., 2008; Woltering, 2010).

**Table 1:** **Proteinases related to PCD**.

**Proteinases**	**Organisms**	**Types**	**Substrate specificity (residue at P_1_ position)**	**Cleavable caspase substrates**
VPE/AEP/legumain	Plants and animals	Cysteine proteinase	Asparagine, aspartic acid	YVAD (caspase-1 substrate)
26S proteasome β1 subunit	Eukaryotes	Threonine proteinase	Glutamic acid, aspartic acid	DEVD (caspase-3 substrate)
Saspase	Plants	Serine proteinase	Aspartic acid	VKMD (caspase-6 substrate), IETD (caspase-8 substrate)
Phytaspase	Plants	Serine proteinase	Aspartic acid	VEID (caspase-6 substrate)
Metacaspase	Plants, Fungi, and protists	Cysteine proteinase	Arginine, lysine	

**Figure 1:**
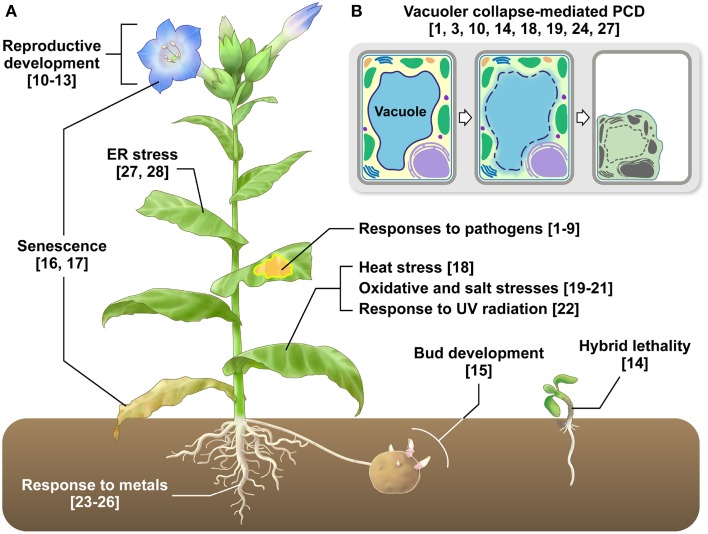
**VPE functions in various types of plant PCD. (A)** VPE-mediated PCD occurs in almost all plant cells and tissues and is involved in developmental processes and responses to biotic and abiotic stresses. Refer to Table 1 for further information. The numbers are reference numbers in Table 1. **(B)** VPE-mediated PCD involves the collapse of vacuolar membranes, which allows vacuolar hydrolytic enzymes to be discharged into the cytosol, resulting in cell death. The numbers are reference numbers in Table 1.

VPE was originally discovered as a cysteine proteinase responsible for the maturation of seed storage proteins in maturing pumpkin seeds, and it was named after its role in the proteolytic processing of various vacuolar proteins (Hara-Nishimura and Nishimura, 1987; Hara-Nishimura et al., 1991). The primary structure of VPE was deduced from the *VPE* cDNA of castor bean (Hara-Nishimura et al., 1993). The molecular characterization of the VPE of *Arabidopsis* showed that VPE is expressed not only in seeds but also in vegetative organs (Hara-Nishimura et al., 1998; Yamada et al., 2005): αVPE and γVPE mainly in vegetative organs (Kinoshita et al., 1995a), βVPE in embryos (Kinoshita et al., 1995b), and δ VPE specifically and transiently in the two cell layers of the seed coat at an early stage of seed development (Nakaune et al., 2005). Genome databases showed that VPE homologs are widely distributed in land plants, from moss (*Physcomitrella patens*) and fern (*Ceratopteris richardii*) to seed plants. In this review, we focus on recent advances in the understanding of the role of VPE in plant PCD. VPE functions in various types of plant PCD are summarized in Table 2 and Figure 1.

**Table 2:** **VPE functions in plant PCD and the related processes**.

**Species**	**Tissues**	**PCD inducer**	**Description**	**References**
*Nicotiana benthamiana*	Leaf	Virus	Vacuolar collapse-mediated PCD; Suppression of PCD and increased viral proliferation in *VPE*-silenced line	Hatsugai et al., 2004, [1]
*Arabidopsis thaliana*	Leaf	Virus, bacteria, fungi	Partial PCD suppression and increased susceptibility to virus and fungi in γ*vpe* mutant	Rojo et al., 2004, [2]
*Nicotiana benthamiana*	Leaf	AtCNGC11/12 overexpression	Vacuolar collapse-mediated PCD; Suppression of PCD in *VPE*-silenced line and by caspase-1 inhibitors	Urquhart et al., 2007, [3]
*Malus* spp. (apple cultivars)	Leaf	Bacteria	Upregulation of *VPE* gene	Iakimova et al., 2013, [4]
*Arabidopsis thaliana*	Leaf	Oomycete	Reduction of oomycete sporulation in *vpe*-null mutant (compatible interactions presumably independent of PCD); Increase of γVPE activity during oomycete infection	Misas-Villamil et al., 2013, [5]
*Nicotiana benthamiana*	Leaf	Bacterial elicitor	Suppressions of PCD and elicitor-induced stomatal closure in *VPE*-silenced line	Zhang et al., 2010, [6]
*Nicotiana tabacum*	Suspension cultured cell	Oomycete elicitor	PCD suppression by caspase-1 inhibitors; Upregulation of *VPE* genes	Gauthier et al., 2007, [7]
*Arabidopsis thaliana*	Leaf	Fungal toxin	PCD suppressions in *vpe*-null mutant and by inhibitors of VPE and caspase-1; Inhibition of VPE activity by recombinant p35 protein	Kuroyanagi et al., 2005, [8]
*Nicotiana umbratica*	Leaf	Fungal toxin	PCD suppression in *VPE*-silenced line	Mase et al., 2012, [9]
*Arabidopsis thaliana*	Seed coat	Developmental	PCD delay in the two cell layers of inner integument of δ vpe mutant; δ *VPE*-gene upregulation and δ VPE-protein induction	Nakaune et al., 2005, [10]
*Hordeum vulgare*	Pericarp	Developmental	Upregulation of *VPE* gene	Radchuk et al., 2010, [11]
*Hordeum vulgare*	Nucellus	Developmental	Upregulation of *VPE* gene with the increase of caspase-like activity	Tran et al., 2014, [12]
*Arabidopsis thaliana*	Circular-cell clusters of anthers	Developmental	Upregulation of *γVPE* gene	Hara-Nishimura, 2012, [13]
*Nicotiana gossei and Nicotiana tabacum* F1 *hybrid*	Seedling	Postzygotic incompatibility	Vacuolar collapse-mediated PCD; Suppression of PCD by caspase-1 inhibitor; Increase of VPE activity	Mino et al., 2007, [14]
*Solanum tuberosum*	Tuber apical bud meristem	Developmental	PCD suppression by caspase-1 inhibitor; Increase of VPE activity	Teper-Bamnolker et al., 2012, [15]
*Arabidopsis thaliana*	Leaf, lateral root	Senescence, developmental	Upregulation of *αVPE* and *γVPE* genes	Kinoshita et al., 1999, [16]
*Nicotiana tabacum*	Petal	Senescence	Upregulation of *VPE* genes	Muller et al., 2010, [17]
*Nicotiana tabacum*	Suspension cultured cell	Heat stress	Vacuolar collapse-mediated PCD; Suppression of PCD in *vpe*-null mutant; *γVPE*-gene upregulation and increase of VPE activity	Li et al., 2012, [18]
*Oryza sativa*	Leaf	Hydrogen peroxide stress	Vacuolar collapse-mediated PCD; PCD suppression by reduction of *VPE* gene expression in Bcl-2 overexpressor	Deng et al., 2011, [19]
*Oryza sativa*	Leaf	salt stress	PCD suppression by reduction of *VPE* gene expression in Bcl-2 overexpressor	Deng et al., 2011; Kim et al., 2014, [20]
*Arabidopsis thaliana*	Suspension cultured cell	Ozone exposure	Upregulation of *γVPE* genes	Kadono et al., 2010, [21]
*Arabidopsis thaliana*	Leaf protoplast	Ultraviolet radiation	PCD suppression by caspase-1 inhibitor and in p35 overexpressor	Danon et al., 2004, [22]
*Lycopersicon esculentum*	Suspension cultured cell	Aluminum	PCD suppression by caspase-1 inhibitor	Yakimova et al., 2007, [23]
*Nicotiana tabacum*	Suspension cultured cell	Aluminum	Vacuolar collapse-mediated PCD; Suppression of PCD by caspase-1 inhibitor; VPE-gene upregulation and increase of VPE activity	Kariya et al., 2013, [24]
*Nicotiana tabacum*	Root	Aluminum	PCD suppression by reduction of VPE gene expression in Ced-9 overexpressor	Wang et al., 2009, [25]
*Lycopersicon esculentum*	Suspension cultured cell	Cadmium	PCD suppression by caspase-1 inhibitor	Yakimova et al., 2006, [26]
*Arabidopsis thaliana*	Leaf	ER stress	Vacuolar collapse-mediated PCD; Suppression of PCD in *vpe*-null mutant; Increase of VPE activity and caspase-1-like activity	Qiang et al., 2012, [27]
*Glycine max*	Leaf protoplast	ER stress	Upregulation of *VPE* gene; Identification of two transcription factors for *VPE* gene expression	Mendes et al., 2013, [28]

## VPE has a caspase-1-like activity

VPE is a vacuole-localized cysteine proteinase responsible for the maturation and activation of vacuolar proteins, which are synthesized on the endoplasmic reticulum (ER) as a proprotein precursor and are then transported to vacuoles (Hara-Nishimura and Nishimura, 1987; Hara-Nishimura et al., 1991). VPE itself is also synthesized as an inactive proprotein precursor. The proprotein precursor of VPE is self-catalytically converted into the active mature form and no other factor is necessary for activating VPE molecules (Hiraiwa et al., 1999; Kuroyanagi et al., 2002). Therefore, VPE is an initiator of the vacuolar-processing system. The pleiotropic functions of the VPE family have been reviewed extensively (Yamada et al., 2005). The self-catalytic conversion of the inactive precursor protein into functional VPE resembles the processing and activation of caspase 1 (Hiraiwa et al., 1999; Nicholson, 1999; Kuroyanagi et al., 2005).

VPEs cleave peptide bonds at the C-terminal sides of not only asparagine residues but also aspartic acids exposed on the surface of proprotein precursors to generate the respective mature proteins (Hara-Nishimura and Nishimura, 1987; Hara-Nishimura et al., 1991, 1993; Becker et al., 1995; Hara-Nishimura, 1998). VPE activity toward a synthetic VPE substrate, benzyloxycarbonyl-AAN-MCA, in virus-infected tobacco plants is inhibited by not only the VPE inhibitor ESEN-CHO but also caspase-1 inhibitors (Hatsugai et al., 2004). In *VPE*-silenced *Nicotiana benthamiana* plants, the reduced VPE activity parallels the reduction of caspase-1-like activity (Hatsugai et al., 2004). In addition, *Arabidopsis* γVPE binds to caspase-1 inhibitors that block the self-maturation of this enzyme and the activation of its downstream enzyme (Rojo et al., 2004). Further experiments showed that an *Arabidopsis vpe*-null mutant, which lacks all four *VPE* genes (*αVPE*, *βVPE*, *γVPE*, and *δ VPE*) in the genome, shows neither VPE activity nor caspase-1-like activity (Kuroyanagi et al., 2005). In addition, recombinant γVPE recognized a VPE substrate with a *K*_m_ = 30.3 μ M and a caspase-1 substrate with a *K*_m_ = 44.2 μ M but not a caspase-3 substrate (Kuroyanagi et al., 2005). Thus, VPE recognizes aspartic acid when it is part of the YVAD sequence of a caspase-1 substrate, but does not necessarily recognize other aspartic acid residues, such as the DEVD sequence of a caspase-3 substrate.

**Figure 2:**
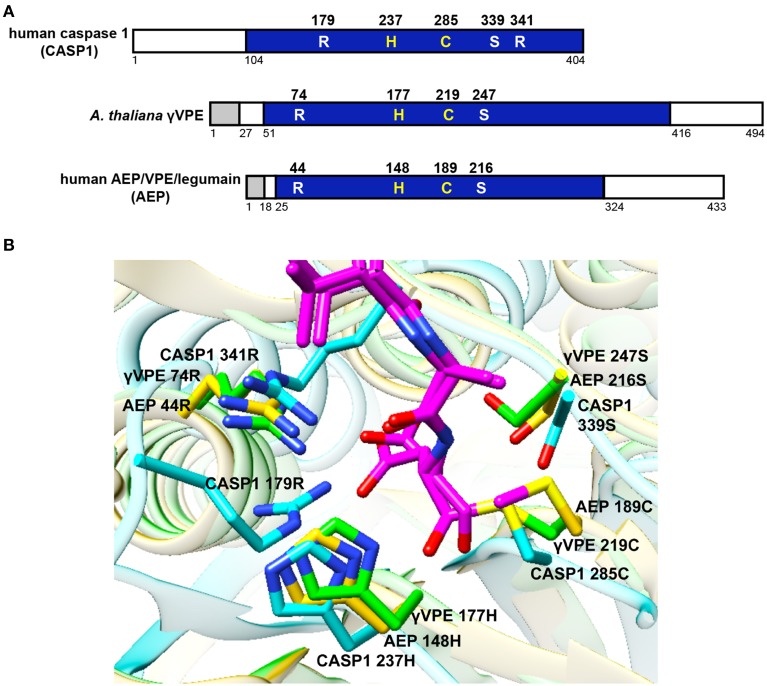
**Comparison of VPE with caspase 1. (A)** Primary structural organizations of the precursor proteins of human caspase 1 (CASP1), *Arabidopsis* γVPE, and human AEP/VPE/legumain (AEP). The γVPE and AEP precursors have a signal peptide (gray boxes) at the N-termini. The proprotein precursors of γVPE, AEP, and CASP1 have cleavable propeptide (open boxes). After the removal of the propeptides, proprotein precursors are converted into the respective mature enzymes (blue boxes). Shown are five essential amino acid residues forming the substrate pocket of CASP1 and their corresponding residues of γVPE and AEP, which are members of the VPE family. His237 and Cys285 form the catalytic dyad of CASP1, whereas His177 and Cys219 form the catalytic dyad of γVPE. Three essential amino acids (Arg179, Ser339, and Arg341) form the substrate-binding pocket of CASP1. **(B)** Predicted 3D structures of the substrate pockets of CASP1, γVPE, and AEP together with the substrate peptide YVAD (magenta) using the program Phyre2 (http://www.sbg.bio.ic.ac.uk/phyre2/). Note that Arg74, His177, Cys219, and Ser247 of γVPE correspond to Arg341, His237, Cys285, and Ser339 of CASP1, respectively. The backbone amino acids of CASP1, γVPE, and AEP are shown in light blue, green, and orange, respectively. Oxygen, red; nitrogen, blue; sulfur, yellow.

The similarity of the substrate specificity between VPE and caspase 1 is consistent with several structural similarities between the two enzymes, such as similar substrate pockets and similar active sites (Stennicke and Salvesen, 1998; Earnshaw et al., 1999; Hara-Nishimura et al., 2005; Hatsugai et al., 2006). Figure 2A shows essential amino acid residues forming the substrate pocket of human caspase 1 and the corresponding residues of *Arabidopsis* γVPE and human AEP/VPE/legumain, which are conserved in Opisthokonta. Our predictions of three-dimensional (3D) structures reveal an interesting feature of the enzymes (Figure 2B). Surprisingly, γVPE Arg74, which has been thought to correspond to caspase-1 Arg179, accurately overlaps with caspase-1 Arg341. Two guanido groups of Arg341 and Arg179 of caspase 1 make strong affinity with the carboxylate group of the Asp residue in the substrate peptide YVAD (Nicholson, 1999). On the other hand, γVPE has only one guanido group of Arg74, which makes the substrate pocket of γVPE less positively charged than that of caspase 1. This is consistent with the fact that γVPE has broader substrate specificity toward Asp and Asn, while caspase 1 has narrow substrate specificity toward Asp.

## VPE roles in developmental PCD

### Reproductive development

In angiosperm seeds, the embryo and endosperm are surrounded by the seed coat. The *Arabidopsis* seed coat consists of two integuments, the outer and inner, of maternal tissues, and multiple cell layers of these integuments develop after fertilization, resulting in the specialized structures of the seed coat. During the early stage of seed development, δ VPE has caspase-1-like activity and is specifically expressed in two cell layers of the inner integuments of the *Arabidopsis* seed coat (Nakaune et al., 2005). This tissue undergoes PCD in its early stages, thereby reducing its thickness. In a δ *VPE*-deficient mutant, however, PCD is delayed and the inner integuments remain thick throughout embryogenesis (Nakaune et al., 2005). This indicates that δ VPE is responsible for the PCD of limited cell layers during the formation of the seed coat.

In barley (*Hordeum vulgare*), seven VPE homologs (HvVPEs) have been identified and some may be involved in PCD during the development of maternal seed tissues, including the nucellus and pericarp (Linnestad et al., 1998; Radchuk et al., 2010; Julian et al., 2013). HvVPE4, which is weakly similar to *Arabidopsis* δ VPE, is exclusively expressed in the deteriorating pericarp associated with apoptotic DNA degradation. This correlative evidence suggested that HvVPE4 is involved in the PCD of the pericarp (Radchuk et al., 2010). HvVPE2a, known as nucellain, is reported to be localized in nucellar cell walls that degenerate in developing cereal grains (Linnestad et al., 1998; Dominguez et al., 2001). HvVPE2a may play a role in the processing and/or turnover of cell wall proteins. Further evidence supporting the involvement of HvVPE2a in nucellar PCD was provided by reports of an increased caspase-1-like activity in the nucellus and nucellar projection during the development of maternal seed tissues in barley (Tran et al., 2014). Additional genetic and biochemical investigations are required to validate the contribution of HvVPE to PCD.

### Hybrid lethality

Hybrid lethality is a common post-zygotic incompatibility and is associated with PCD (Bomblies and Weigel, 2007). The interspecific F1 hybrid of *Nicotiana gossei* and *Nicotiana tabacum* exhibits hybrid lethality at the seedling stage. The cell death in the hybrid seedling is proceeded by vacuolar collapse (Mino et al., 2007). The vacuolar collapse and cell death were suppressed by the inhibition of VPE activity, and there was a correlation between VPE activity and the breakdown of the vacuolar membrane. These results suggest that this protease is involved in the cell death underlying hybrid lethality.

### Bud development and senescence

Furthermore, in the developmental program, VPE is associated with PCD in tuber apical bud meristems and the release of apical dominance in potato tubers (Teper-Bamnolker et al., 2012). VPE may also be related to petal and leaf senescence (Kinoshita et al., 1999; van Doorn and Woltering, 2008; Muller et al., 2010). Promoter-GUS analyses showed the up-regulation of *αVPE* and *γVPE* in dying cortex cells located next to the emerging lateral root (Kinoshita et al., 1999) and in dying circular-cell clusters of anthers during the later stage of pollen development (Hara-Nishimura, 2012), respectively.

## VPE roles in biotic stimuli-induced PCD

### Responses to pathogens

Plants are continuously challenged by a wide variety of pathogens, such as viruses, bacteria, fungi, and oomycetes. In most cases, however, the spread of disease is limited by plant immune responses, including the hypersensitive response (HR), which is characterized by a rapid and localized PCD known as hypersensitive cell death (Greenberg, 1997). The HR is controlled by multiple signal transduction pathways that are initiated upon the recognition of a pathogen avirulence (Avr) factor by a plant resistance (R) gene product (Dangl and Jones, 2001; Jones and Dangl, 2006). Caspase peptide inhibitors suppress the HR in response to infection with an avirulent *Pseudomonas syringae* pv *phaseolicola* strain (del Pozo and Lam, 1998). In addition, caspase-like activity was detected in tobacco plants in response to tobacco mosaic virus (TMV) (del Pozo and Lam, 1998). This work was the first report of the involvement of caspase-like activity in plant PCD.

Studies using a virus-induced gene silencing strategy provided evidence that VPE is a proteinase exhibiting caspase-1-like activity and that, by controlling vacuolar rupture in *N. benthamiana*, it was essential for TMV-induced hypersensitive cell death (Hatsugai et al., 2004). The vacuolar collapse has been proposed to be the crucial event in plant cell death (Jones, 2001). An ultrastructural analysis and a viability assay showed that TMV-induced cell death was preceded by the disintegration of vacuolar membranes and that membrane disintegration continued, resulting in complete vacuolar collapse (Hatsugai et al., 2004). In contrast, *VPE*-silenced plants did not undergo vacuolar membrane disintegration or cell death (Hatsugai et al., 2004). These silenced plants also failed to show any PCD hallmarks, such as DNA fragmentation, when challenged with TMV. This observation suggests that VPE functions as a key molecule in PCD triggered by vacuolar collapse. Although the *VPE* deficiency does not interfere with the induction of defense genes, virus proliferation is markedly increased in the plants. These observations support the idea that PCD during the HR is critical for the removal of biotrophic pathogens, whose growth depends on the living host tissues (Greenberg and Yao, 2004).

The chimeric *Arabidopsis* cyclic nucleotide-gated ion channels, AtCNGC11 and AtCNGC12, act as positive regulators of *R* gene-mediated resistance responses (Yoshioka et al., 2006; Moeder et al., 2011). AtCNGC11/12 can induce hypersensitive cell death when transiently expressed in *N. benthamiana* (Urquhart et al., 2007). A microscopic analysis of dying cells revealed that the cell death exhibits morphological and biochemical features of PCD, and involves vacuolar membrane rupture and vacuole collapse (Urquhart et al., 2007). Interestingly, in *VPE*-silenced plants, the development of cell death induced by AtCNGC11/12 was much slower and weaker compared with in control plants (Urquhart et al., 2007). These results indicated the involvement of VPE in AtCNGC11/12-induced cell death.

Recently, it was suggested that VPE is related to the HR induced by *Erwinia amylovora* in apple leaves (Iakimova et al., 2013). In addition, a role for VPE during compatible interactions between *Arabidopsis* and the obligate biotrophic oomycete pathogen *Hyaloperonospora arabidopsidis* has been demonstrated, but is presumably independent of PCD (Misas-Villamil et al., 2013). Using an activity-based probe for *Arabidopsis* VPE, the γVPE activity was shown to increase during an *H. arabidopsidis* infection. Interestingly, the *Arabidopsis vpe*-null mutant decreased *H. arabidopsidis* sporulation, indicating that VPEs are beneficial for *H. arabidopsidis* pathogenicity. This suggested that, as an obligate biotrophic pathogen, *H. arabidopsidis* takes advantage of the increased VPE activity in the host cells. The enhanced resistance is only partial in the *γvpe* single mutant, suggesting that other VPEs also contribute to *H. arabidopsidis* sporulation.

Three elicitors, harpin, Nep1, and boehmerin, which are produced by bacteria, fungi and oomycete, respectively, induce hypersensitive cell death in *N. benthamiana* (Wei et al., 1992; Wang et al., 2003; Gijzen and Nurnberger, 2006). A study examined whether VPE contributed to elicitor-induced cell death (Zhang et al., 2010). After infiltration with each of the three elicitors, only harpin-induced cell death was compromised in *NbVPE1a*- and *NbVPE1a/1b*-silenced plants, suggesting that *NbVPE1a* contributes to harpin-induced cell death (Zhang et al., 2010). However, hypersensitive cell death was not impaired in the gene-silenced plants in response to Nep1 and boehmerin. This was consistent with the hypersensitive cell death triggered by Nep1 in *Phytophthora sojae* not requiring caspase-like activity (Qutob et al., 2006). These results suggest that the molecular mechanism for hypersensitive cell death triggered by harpin differs from that triggered by Nep1 or boehmerin. Additionally, VPE may be associated with the hypersensitive cell death triggered by the oomycete elicitor, cryptogein (Gauthier et al., 2007).

Some necrotrophic pathogens secrete mycotoxins to kill host cells and promote their own growth in susceptible host plants (Walton, 1996; Markham and Hille, 2001). A fungal pathogen, *Fusarium moniliforme*, produces fumonisin B1 (FB1) and causes disease symptoms in maize. FB1 inhibits ceramide synthase, which is responsible for sphingolipid biosynthesis (Wang et al., 1991), resulting in PCD in *Arabidopsis* plants. Kuroyanagi et al. (2005) showed that FB1-induced cell death was accompanied by the disintegration of vacuolar membranes and DNA fragmentation, followed by lesion formation (Kuroyanagi et al., 2005). The features of FB1-induced cell death were completely abolished by the caspase-1 inhibitor and in the *Arabidopsis* quadruple *vpe*-null mutant (Kuroyanagi et al., 2005). The *γVPE* expression was also increased after *Botrytis cinerea*, a necrotrophic fungi infection (Rojo et al., 2004).

Additionally, the *γvpe* single mutant more severely suppressed lesion formation than the other single mutants (*αvpe*, *βvpe*, and *δ vpe*), although the suppression was not as strong as it was in the *vpe*-null mutant plants. The other VPEs possibly compensate for the lack of γVPE in *γvpe* leaves. γVPE is the most essential of the four VPE homologs for FB1-induced cell death in *Arabidopsis* leaves. The compensation among VPEs is not unique to PCD. It is also found in seed storage protein processing, where αVPE and γVPE compensate for the lack of *β* VPE in the *βvpe* mutant (Shimada et al., 2003).

The AAL-toxin produced by the fungus *Alternaria alternata* f. sp. *lycopersici* is a chemical congener of FB1 (Nelson et al., 1993) and causes disease symptoms in susceptible tomatoes (Wang et al., 1996) and some *Nicotiana* species lacking the *Alternaria stem canker gene 1* (Brandwagt et al., 2000). AAL-toxin-induced cell death was suppressed in tomato by overexpressing the anti-apoptotic baculovirus p35 (Lincoln et al., 2002), which is a caspase inhibitor. The recombinant p35 inhibited VPE activity *in vitro* (Kuroyanagi et al., 2005), suggesting that AAL-toxin-induced cell death is mediated by VPE. In fact, the cell death induced by the AAL-toxin was compromised in *VPE*-silenced *Nicotiana umbratica* plants (Mase et al., 2012). Additionally, the pathogenicity of *A. alternata* f. sp. *lycopersici* was abolished in VPE-silenced *N. umbratica* plants (Mase et al., 2012). These results suggested that VPE is involved in fungal toxin-induced cell death in plants.

### ER stress

The ER is an organelle in which secretory and membrane proteins are correctly folded and assembled by chaperones. When these processes do not function properly, unfolded or misfolded proteins accumulate inside the ER, causing ER stress. Such ER stress triggers the unfolded protein response (UPR), which adjusts the protein-folding capacity to the needs of the cell, to avoid cell damage. However, prolonged ER stress eventually overwhelms the cellular protective mechanisms and ultimately results in PCD. In animals, ER stress-induced PCD involves the activation of caspase (Rasheva and Domingos, 2009). In plants, there have been several reports implicating caspase-like activities in ER stress-induced PCD (Cai et al., 2014).

Qiang et al. (2012) showed, using a cytological analysis of mutualistic interactions between *Arabidopsis* and *Piriformospora indica*, that caspase-1 and VPE activities regulated ER stress-induced PCD. The mutualistic fungi *P. indica* disturbs the UPR, which eventually leads to the death of root cells. An ultrastructural analysis showed that *P. indica* colonization was associated with ER swelling, which was followed by tonoplast rupture. In a *vpe*-null mutant, the tonoplast rupture was not detected and PCD was compromised, although ER swelling occurred. These results indicated that ER stress-induced vacuole-mediated cell death is dependent on VPE. VPE might additionally function downstream of UPR in the ER stress-induced PCD process. A recent report supported this hypothesis. Two NAC transcription factors, GmNAC30 and GmNAC81, that induce PCD downstream of osmotic and ER stresses, are able to interact with each other in a synergistic manner to directly activate *VPE* gene expression (Mendes et al., 2013).

## VPE roles in abiotic stress-induced PCD

### Heat stress

Heat shock triggers PCD, with apoptotic features including cell shrinkage, chromatin condensation, and DNA fragmentation (Tian et al., 2000; Vacca et al., 2006). In heat-treated tobacco BY-2 cells, cell death has been reported to be prevented by both caspase-1 and caspase-3 inhibitors (Vacca et al., 2006), but the link between caspase-1-like and caspase-3-like activities and the signaling pathway leading to PCD remains to be investigated. Recent work using the *Arabidopsis vpe*-null mutant showed that VPE exhibited caspase-1-like activity in heat-treated leaves and promoted both vacuolar disruption and activation of caspase-3-like activity (Li et al., 2012). This may provide the first evidence for the participation of VPE in the activation of a downstream caspase-3-like activity. In further studies, MAP kinase 6 (MPK6) activity was increased after heat shock treatment, and experiments with inhibitors and mutants suggested that MPK6 was responsible for the γVPE activation and the subsequent execution of PCD. These results suggest that the activation of γVPE was mediated by MPK6 and played an important role in heat shock-induced PCD in *Arabidopsis* (Li et al., 2012).

### Oxidative and salt stresses

H_2_O_2_ is an important signaling molecule that regulates stress-induced plant PCD (Gechev and Hille, 2005). In rice, five *VPE* (*OsVPE*) genes were found in the genome (Christoff et al., 2014), and OsVPE2 and OsVPE3 may be involved in the H_2_O_2_-induced PCD. The expression levels of *OsVPE2* and *OsVPE3* were up-regulated during H_2_O_2_ stress. Furthermore, the H_2_O_2_-induced enhancement of *OsVPE2* and *OsVPE3* expression levels were significantly suppressed, as was the cell death associated with vacuolar rupture in rice transgenic lines overexpressing Bcl-2, which is a potent inhibitor of human apoptosis (Deng et al., 2011). In addition, the PCD caused by high salt stress was also effectively suppressed by Bcl-2, and the salt-induced expression levels of *OsVPE2* and *OsVPE3* were markedly inhibited in Bcl-2-overexpressing rice lines (Deng et al., 2011; Kim et al., 2014). These results suggested that *OsVPE2* and *OsVPE3* are involved in H_2_O_2_- and salt stress-induced PCD, and also that Bcl-2 inhibits the induced PCD by suppressing the transcriptional activation of *OsVPEs* in rice. H_2_O_2_- and salt stress-induced PCD may share a common pathway that is suppressed by Bcl-2, thereby inhibiting the transcriptional activation of *OsVPE* in rice. Additional experiments, including the study of the effect of H_2_O_2_ andsalt stress on VPE-deficient lines, will be necessary to test this hypothesis.

The air pollutant ozone (O_3_) triggers H_2_O_2_ production and subsequently causes visible lesion formations on leaves, which is similar to the pathogen-induced HR. The O_3_-induced cell death requires caspase-like activities (Pasqualini et al., 2003; Kangasjärvi et al., 2005; Overmyer et al., 2005). The *γVPE* expression level was increased early after O_3_ exposure to *Arabidopsis* suspension cells (Kadono et al., 2010). The O_3_-induced up-regulation of *γVPE* was suppressed efficiently by an NADPH oxidase inhibitor (diphenyleneiodonium) and anion channel blockers (9-anthracen carboxylic acid and glibenclamide), suggesting that anion channel activation and H_2_O_2_ production are involved in the signaling pathway leading to a transcriptional regulation of *γVPE* for the O_3_-induced cell death.

### Response to ultraviolet (UV) radiation

VPE has been implicated in the PCD induced by UV radiation (Danon et al., 2004). UV-induced DNA fragmentation and cell death were suppressed by caspase-1 and caspase-3 inhibitors. Furthermore, the UV-induced cell death associated with DNA fragmentation was prevented in *Arabidopsis* protoplasts overexpressing anti-apoptotic baculovirus p35. Because the recombinant p35 inhibited VPE activity *in vitro* (Kuroyanagi et al., 2005), UV-induced cell death might be mediated by VPE, as is the case with toxin-induced cell death (Lincoln et al., 2002).

### Response to metals

Aluminum is known to be toxic to plants as well as humans, and it inhibits cell division and root elongation, eventually resulting in cell death (Kochian, 1995). Aluminum-induced cell death is accompanied by typical apoptotic features, such as nuclear and DNA fragmentation and cytoplasmic condensation. In tomato suspension-cultured cells, cell death was abolished by a broad-range of caspase inhibitors (Yakimova et al., 2007). This was the first evidence showing the involvement of a proteinase possessing caspase-like activity in aluminum-induced PCD.

A recent study reported that a caspase-1 inhibitor, Ac-YVAD-CHO, prevented the loss of plasma membrane integrity caused by exposure to aluminum in tobacco suspension-cultured BY-2 cells (Kariya et al., 2013). Time-course experiments indicated that the VPE activity increased after the aluminum treatment, which might cause a loss of plasma membrane integrity. In addition, fluorescence microscopic observations of a transgenic cell line expressing a tonoplast-localized GFP-AtVam3p demonstrated that vacuolar rupture occurred prior to cell death in aluminum-treated cells. These results suggested that VPE-mediated vacuolar collapse was a key factor leading to aluminum-induced PCD in plants. However, how aluminum triggers an increase in VPE activity remains to be elucidated.

Additionally, a previous study revealed that the apoptotic suppressor, Ced-9, effectively inhibited aluminum-induced PCD and promoted aluminum tolerance in plants, possibly by inhibiting aluminum-induced VPE activity (Wang et al., 2009). It is likely that conserved negative regulators of PCD are involved in the integrated regulation of aluminum-induced PCD by an unidentified mechanism (Wang et al., 2009).

The caspase-1-like and VPE activities may also be associated with the PCD induced by cadmium, which is a widespread heavy metal pollutant (di Toppi and Gabbrielli, 1999). In tomato suspension-cultured cells, cadmium induced cell death within 24 h in a concentration-dependent manner. The cadmium-induced cell death was almost completely abolished by the caspase-1 inhibitor Ac-YVAD-CMK and the broad-range caspase inhibitor Z-Asp-CH_2_-DCB. The cell death kinetics and morphological features were comparable to the effects of aluminum (Yakimova et al., 2006). These results suggest that cadmium-induced cell death may also proceed through a VPE-mediated vacuolar system.

## VPE-dependent activation of defense proteins

VPE post-translationally processes precursor proteins to produce various functional proteins in vacuoles. VPE cleaves the multiple Asn-Gln bonds of the single precursor protein PV100 to produce different functional proteins, including a proteinase inhibitor, cytotoxic peptides and a storage protein, in pumpkin seeds (Yamada et al., 1999). Interestingly, VPE catalyzes not only the peptide-bond-cleavage reaction but also the peptide-ligation or transpeptidation reaction. The latter reaction generates a backbone-cyclized protein, cyclotide kalata B1, from a precursor protein in sunflower seeds (Saska et al., 2007). Kalata B1 functions in defense against insect pests by inhibiting their growth (Jennings et al., 2001). Thus, VPE produces defense proteins from the inactive precursors that have cleavable Asn residues. The defense proteins also include a proteinase inhibitor of tomato leaves (Graham et al., 1985), a proteinase inhibitor of tobacco stigmas (Atkinson et al., 1993), and a basic chitinase of tobacco leaves (Sticher et al., 1993). Hence, VPE plays a key role in generating active defense proteins against pathogens.

## Animal AEP/VPE/legumain

VPE family members are widely distributed in plants and animals. Mammalian VPE homologs are also referred as to asparaginyl endopeptidase (AEP/VPE/legumain), because of the substrate specificity toward asparaginyl bonds (Chen et al., 1997). The crystal structure of human AEP was recently reported to show the asparagine-specific endopeptidase activity (Dall and Brandstetter, 2013) (Figure 2B). A study using AEP-null mice showed that AEP is required for the maturation of lysosomal proteinases (cathepsins B, L, and H) and that AEP has a critical role in the degradation of cellular materials in the endosomes/lysosomes of kidney cells (Shirahama-Noda et al., 2003). The AEP-null mice also provided evidence that AEP is involved in neuronal cell death, whereby AEP appears to degrade a DNase inhibitor (SET), which is a caspase substrate, and trigger DNA damage in the brain (Liu et al., 2008). Based on these observations, a similar VPE/AEP-dependent PCD mechanism appears to function in plants and animals; processing the vacuole/lysosome degradation enzymes and then collapsing membranes to leak degradation enzymes into the cytosol during PCD.

## Concluding remarks

VPE is a plant counterpart of caspase 1 and is involved in the execution of a variety of plant PCDs. However, the subcellular localization of these enzymes is different: caspase 1 is a cytosolic enzyme and VPE is a vacuolar enzyme. This implies that the death mechanisms are different between plants and animals. In animal apoptosis, while caspases certainly play a central role, an increasing body of evidence suggests that lysosomal proteases, such as cathepsin B, are involved in the initiation and/or execution of the apoptotic program (Guicciardi et al., 2004). The lysosomal release of cathepsin B into the cytosol is capable of triggering mitochondrial dysfunction with subsequent caspase activation and cellular demise. The lysosomal cathepsin B is processed by AEP (Shirahama-Noda et al., 2003). Plant PCD is accompanied by the up-regulation of a variety of vacuolar hydrolytic enzymes (Fukuda, 2004). VPE could mediate the initial activation of some of these vacuolar enzymes, which then degrade the vacuolar membrane and initiate the proteolytic cascade leading to PCD. These findings suggest that the release of VPE/AEP-dependently-activated enzymes from vacuoles/lysosomes is a common event in both plant and animal PCD. The mechanism by which VPE controls vacuolar rupture is still unclear and could be addressed using the variety of PCD systems that have been described herein.

### Conflict of interest statement

The authors declare that the research was conducted in the absence of any commercial or financial relationships that could be construed as a potential conflict of interest.
